# Correction: Protein biomarkers predictive for response to anti-EGFR treatment in RAS wild-type metastatic colorectal carcinoma

**DOI:** 10.1038/s41416-018-0130-x

**Published:** 2018-06-14

**Authors:** Astrid Lièvre, Bérèngere Ouine, Jim Canet, Aurélie Cartier, Yael Amar, Wulfran Cacheux, Odette Mariani, Rosine Guimbaud, Janick Selves, Thierry Lecomte, Serge Guyetant, Ivan Bieche, Frédérique Berger, Leanne de Koning

**Affiliations:** 1 Service des maladies de l’appareil digestif, CHU Pontchaillou, 2 rue Henri Le Guilloux, Rennes Cedex, 09 35033 France; 2 Faculté de médecine, Université Rennes 1, 2 Avenue du Prof. Léon Bernard, Rennes, 35043 France; 3 Inserm ER440-Oncogenesis, Stress and Signaling, Rue Bataille Flandres-Dunkerque, Rennes, 35042 France; 4 Department of Translational Research, Institut Curie, PSL Research University, 26 rue d’Ulm, Paris, 75005 France; 5 Institut Curie, PSL Research University, Unit of Biostatistics, 26 rue d’Ulm, Paris, 75005 France; 6 Department of Medical Oncology, Institut Curie, René Huguenin Hospital, 35 rue Dailly, Saint-Cloud, 92210 France; 7 Department of Genetics, Institut Curie, Unit of Pharmacogenomics, 26 rue d’Ulm, Paris, 75005 France; 8 Biological Resource Center, Institut Curie, PSL Research University, 26 rue d’Ulm, Paris, 75005 France; 9 Centre de Recherche en Cancérologie de Toulouse, Unité Mixte de Recherche, 1037 INSERM—Université Toulouse III, Toulouse, 31062 France; 10 Service d’oncologie médicale, Centre Hospitalier Universitaire de Toulouse, Toulouse, 31059 France; 11 Department of Pathology, Centre Hospitalier Universitaire de Toulouse, Toulouse, 31059 France; 12 Hôpital Trousseau—CHRU de TOURS, Service d’Hépato-Gastro-Entérologie, Tours, 37000 France; 13 UMR CNRS 7292 (GICC), Université François Rabelais, Tours, 37000 France; 14 Hôpital Trousseau—CHRU de TOURS, Service d’Anatomie et Cytologie Pathologiques—Tumorothèque, Tours, 37000 France; 15 Institut Curie, PSL Research University, INSERM U900, 26 rue d’Ulm, Paris, 75005 France

Correction to: Br J Cancer (2018) 117, 1819–1827; 10.1038/bjc.2017.353; published online 12 October 2017

Supplementary Table 1 and the Supplementary Figure Legends were not included when this manuscript was first published. The files are now available here.

## Electronic supplementary material

Supplementary Table 1

Supplementary Figure Legends

