# Supplementary material for: Correction: Protein biomarkers predictive for response to anti-EGFR treatment in RAS wild-type metastatic colorectal carcinoma
Source: Br J Cancer. 2018 Jun 14;119(3):387. doi: 10.1038/s41416-018-0130-x (PMC6079395; doi:10.1038/s41416-018-0130-x)
Supplement: Supplementary file 1 — Supplementary Table 1 [file 41416_2018_130_MOESM1_ESM.docx]

**Supplementary Table 1: Antibodies used for RPPA analyses**

**R: Rabbit, M: Mouse, CST: Cell Signaling Technology, BD: BD Biosciences**

| **Name** | **Species** | **Supplier** | **Reference** |
| --- | --- | --- | --- |
| **Tyrosine kinases / receptors** |  |  |  |
| **EGFR (D38B1)** | R | CST | 4267 |
| **Phospho-EGFR (Tyr 992)** | R | CST | 2235 |
| **Phospho-EGFR (Thr669)** | R | CST | 3056 |
| **Phospho-EGFR (Tyr 1173) (53A5)** | R | CST | 4407 |
| **HER2/ErbB2** | M | Thermo | MA5-14057 |
| **HER2/ErbB2** | M | Lab Vision | MS-1350-P1 (Ab20) |
| **Phospho-HER2/ErbB2 (Tyr1139)** | R | Epitomics | 1991-1; ab53290 |
| **FGF Receptor3 (D2G7E)** | R | CST | 3163 |
| **FGF receptor4** | R | CST | 8562 |
| **HER3/ErbB3 (c-17)** | R | Santa-Cruz | sc-285 |
| **Phospho-Her3/Erbb3 (tyr1289)** | R | CST | 4791 |
| **HER4/ErbB4** | R | Epitomics | 2218-1 |
| **Phospho-HER4 (Tyr1162)** | R | Epitomics | 2295-1 |
| **FAK** | R | CST | 3285 |
| **Phospho-FAK (Tyr861)** | R | Epitomics | 2153-1 |
| **Src (36D10)** | R | CST | 2109 |
| **Phospho-Src (Tyr527)** | R | CST | 2105 |
| **Met** | R | Santa-Cruz | sc-10 (C-12) |
| **Phospho-Met (Tyr1349)** | R | CST | 3133 |
| **IGF-I receptor B** | R | CST | 9750 |
|  |  |  |  |
| **PI3K/Akt pathway** |  |  |  |
| **PI3 Kinase p110 subunit Beta** | R | CST | 3011 |
| **Akt** | R | CST | 9272 |
| **Phospho-Akt (Thr308) (D25E6)** | R | CST | 13038 |
| **Phospho-Akt (Ser473) (193H12)** | R | CST | 4058 |
| **PTEN (D4.3) XP** | R | CST | 9188S |
| **Phospho-PTEN (ser380/Thr382/383)** | R | CST | 9554 |
| **mTOR** | R | Abcam | ab51089 |
| **phospho-mTOR (Ser2448)** | R | Abcam | ab109268 |
| **p70 S6 Kinase** | R | CST | 2708 |
| **Phospho-p70 S6 kinase (Thr421/Ser424)** | R | Upstate (Millipore) | 04-393 |
| **Phospho-p70 S6 Kinase (Thr389)** | R | CST | 9205 |
| **S6 Ribosomal Protein (5G10)** | R | CST | 2217 |
| **Phospho-S6 Ribosomal Protein (Ser235/236)** | R | CST | 2211 |

| **Ras/MAPK pathway** |  |  |  |
| --- | --- | --- | --- |
| **K-RAS** | M | Santa-Cruz | sc-30 (F234) |
| **B-Raf** | R | Santa-Cruz | sc-166 |
| **MEK1/2** | R | CST | 9122S |
| **Phospho-MEK1/2 (Ser217/221)** | R | CST | 9154 |
| **p44/42 MAPK** | R | CST | 9102 |
| **Phospho-p44/42 MAPK (Thr202/Tyr204)** | R | CST | 4377 (197G2) |
|  |  |  |  |
| **Downstream effectors** |  |  |  |
| **4E-BP1** | R | CST | 9452 |
| **Phospho-4E-BP1 (Thr70)** | R | Epitomics | 2250-1 |
| **eIF4E** | R | CST | 9742 |
| **eIF4B** | R | CST | 3592 |
| **Phospho-eIF4B (Ser422)** | R | CST | 3591 |
| **PLC gamma1** | R | CST | 2822 |
| **Phospho-PLC gamma1 (Tyr771)** | R | Epitomics | 2350-1 |
| **Phospho-PLC gamma1 (Tyr783)** | R | CST | 2821 |
|  |  |  |  |
| **Proliferation/Apoptosis** |  |  |  |
| **p53** | R | CST | 9282 |
| **Phospho-p53 (Ser392)** | R | Epitomics | 2326-1 |
| **Phospho-p53 (Ser15)** | R | CST | 9284 |
| **Caspase7** | R | Epitomics | 1032-1 |
| **Cleaved Caspase7 (Asp198)** | R | CST | 9491 |
| **Cleaved Caspase8 (Asp391)** | R | Thermo | MA5-15054 |
| **PARP uncleaved p116** | R | Epitomics | 1077-1 / ab32378 |
| **Cleaved PARP (Asp214) p25** | R | Epitomics | 1051-1 |
| **BAD** | M | BD | 610391 |
| **Bcl2** | R | CST | 2876 |
| **Cyclin D1** | R | Epitomics | 2261-1 |
| **Topoisomerase II alpha** | R | Epitomics | 1826-1 |
| **Phospho-Topoisomerase II a (Thr1343)** | R | Epitomics | 1871-1 / ab52853 |
| **Ki67 (MIB-1)** | M | Dako | M7240 |
| **Cytochrome c (136F3)** | R | CST | 4280 |
|  |  |  |  |
| **Angiogenesis** |  |  |  |
| **VEGF Receptor1 (clone Y103)** | R | Novus | NB110-57643 |
| **Phospho-VEGF Receptor2 (Tyr1175)** | R | Novus | NB100-82260 |
| **PDGFR beta** | R | CST | 3169 |
| **phospho-PDGFR beta (Tyr1021)** | R | CST | 2227 |
|  |  |  |  |
| **Jak/Stat** |  |  |  |
| **Stat1** | R | CST | 9172 |
| **Phospho-Stat1 (Y701)** | R | Abcam | ab109457 |
| **Stat3** | R | CST | 9132 |
| **Phospho-Stat3 (Tyr705) (D3A7)** | R | CST | 9145 |
| **Phospho-Stat3 (Ser727)** | R | CST | 9134 |
| **Jak2 (D2E12)** | R | CST | 3230 |
|  |  |  |  |
| **Protein Kinase C** |  |  |  |
| **PKC alpha** | M | Upstate (Millipore) | 05-154 |
| **Phospho-PKC alpha (Ser657)** | R | Upstate (Millipore) | 06-822 |
| **PKC delta** | R | Epitomics | 2053-1 |
| **Phospho-PKC delta (Thr505)** | R | CST | 9374 |
|  |  |  |  |
| **TGF beta** |  |  |  |
| **TGF-beta I/III (56E4)** | R | CST | 3709 |
| **Smad3 (P84022)** | R | Epitomics | 1735-1 |
| **Phospho-Smad3 (Ser423/425)** | R | Epitomics | 1880-1 |
|  |  |  |  |
| **Wnt/NOTCH** |  |  |  |
| **Beta Catenin (6B3)** | R | CST | 9582 |
| **Phospho-Beta Catenin (Ser675)** | R | CST | 4176 |
| **Notch1** | R | Epitomics | 1935-1 |
| **Cleaved Notch1 (Val1744) (D3B8)** | R | CST | 4147 |
| **GSK3 alpha/beta (0011-A)** | M | Santa-Cruz | sc-7291 |
| **Phospho-GSK3 alpha/beta (Ser21/9)** | R | CST | 9331 |
| **Phospho-GSK3 alpha/beta (Tyr279/216)** | R | Epitomics | 2309-1 |
