# Supplementary material for: Correction: Protein biomarkers predictive for response to anti-EGFR treatment in RAS wild-type metastatic colorectal carcinoma
Source: Br J Cancer. 2018 Jun 14;119(3):387. doi: 10.1038/s41416-018-0130-x (PMC6079395; doi:10.1038/s41416-018-0130-x)
Supplement: Supplementary file 2 — Supplementary Figure Legends [file 41416_2018_130_MOESM2_ESM.docx]

**Supplementary data**

**Supplementary Figure 1**: Hierarchical clustering of the RPPA data shows two distinct groups of samples that are not related to response to therapy (color bar 1), to whether the tumor is right- or left-sided (color bar 2), to the center of origin (color bar 3) or to the type of tumor (color bar 4). Samples are horizontal, antibodies are vertical. White: missing data.

**Supplementary Figure 2**: Kaplan Meier curves of overall survival in right- and left-sided mCRC.

**Supplementary Figure 3**: PI3K pathway activation status in *PIK3CA* wildtype (WT) and mutant (MUT) samples, showing the ratios of Phospho-Akt / Akt (p=0.11), phospho-p70S6K /p70S6K (p=0.14) and phospho-PKCα/ PKCα (p=0.10).
